# *Erratum*: Castellví, M. et al. Pharmacological Modulation of SAMHD1 Activity by CDK4/6 Inhibitors Improves Anticancer Therapy. *Cancers* 2020, *12*, 713

**DOI:** 10.3390/cancers12071728

**Published:** 2020-06-29

**Authors:** Marc Castellví, Eudald Felip, Ifeanyi Jude Ezeonwumelu, Roger Badia, Edurne Garcia-Vidal, Maria Pujantell, Lucía Gutiérrez-Chamorro, Iris Teruel, Anna Martínez-Cardús, Bonaventura Clotet, Eva Riveira-Muñoz, Mireia Margelí, José A. Esté, Ester Ballana

**Affiliations:** 1 IrsiCaixa AIDS Research Institute, Badalona, 08916 Catalonia, Spain; mcnadal89@gmail.com (M.C.); iezeonwumelu@irsicaixa.es (I.J.E.); rbadia@irsicaixa.es (R.B.); egvidal@irsicaixa.es (E.G.-V.); mpujantell@irsicaixa.es (M.P.); lgutierrez@irsicaixa.es (L.G.-C.); bclotet@irsicaixa.es (B.C.); eriveira@irsicaixa.es (E.R.-M.); jaeste@cienciatraducida.com (J.A.E.); 2 Health Research Institute Germans Trias i Pujol (IGTP), Hospital Germans Trias i Pujol, Universitat Autònoma de Barcelona, 08916 Badalona, Spain; efelip@irsicaixa.es (E.F.); iteruel@iconcologia.net (I.T.); amartinezc@igtp.cat (A.M.-C.); mmargeli@iconcologia.net (M.M.); 3 Institut Català d’Oncologia, Badalona, 08916 Catalonia, Spain; 4 Cienciatraducida, 08391 Barcelona, Spain

The authors wish you make the following corrections to this paper [1]. 

One contributor’s name was missing in the original version of the authorship. The original version of the authorship is: 

Marc Castellví ^1,2,†^, Eudald Felip ^2,3,†^, Ifeanyi Jude Ezeonwumelu ^1,2^, Roger Badia ^1,2^, Edurne Garcia-Vidal ^1,2^, Maria Pujantell ^1,2^, Lucía Gutiérrez-Chamorro ^1,2^, Iris Teruel ^2,3^, Anna Martínez-Cardús ^2,3^, Bonaventura Clotet ^1,2^, Eva Riveira-Muñoz ^1,2^, Mireia Margelí ^2,3^ and Ester Ballana ^1,2,^*

and should be replaced with:

Marc Castellví ^1,2,†^, Eudald Felip ^2,3,†^, Ifeanyi Jude Ezeonwumelu ^1,2^, Roger Badia ^1,2^, Edurne Garcia-Vidal ^1,2^, Maria Pujantell ^1,2^, Lucía Gutiérrez-Chamorro ^1,2^, Iris Teruel ^2,3^, Anna Martínez-Cardús ^2,3^, Bonaventura Clotet ^1,2^, Eva Riveira-Muñoz ^1,2^, Mireia Margelí ^2,3^, José A. Esté ^1,4,^ and Ester Ballana ^1,2,^*

The updated “Author Contributions” should be 
