# Supplementary material for: Pharmacological Modulation of SAMHD1 Activity by CDK4/6 Inhibitors Improves Anticancer Therapy
Source: Cancers (Basel). 2020 Mar 18;12(3):713. doi: 10.3390/cancers12030713 (PMC7140116; doi:10.3390/cancers12030713)
Supplement: Supplementary file 1 [file cancers-12-00713-s001.docx]

Supplementary Materials

Pharmacological Modulation of SAMHD1 Activity by CDK4/6 Inhibitors Improves Anticancer Therapy

Marc Castellví, Eudald Felip, Ifeanyi Jude Ezeonwumelu, Roger Badia, Edurne Garcia-Vidal, Maria Pujantell, Lucía Gutiérrez-Chamorro, Iris Teruel, Anna Martínez-Cardús, Bonaventura Clotet, Eva Riveira-Muñoz, Mireia Margelí, Ester Ballana


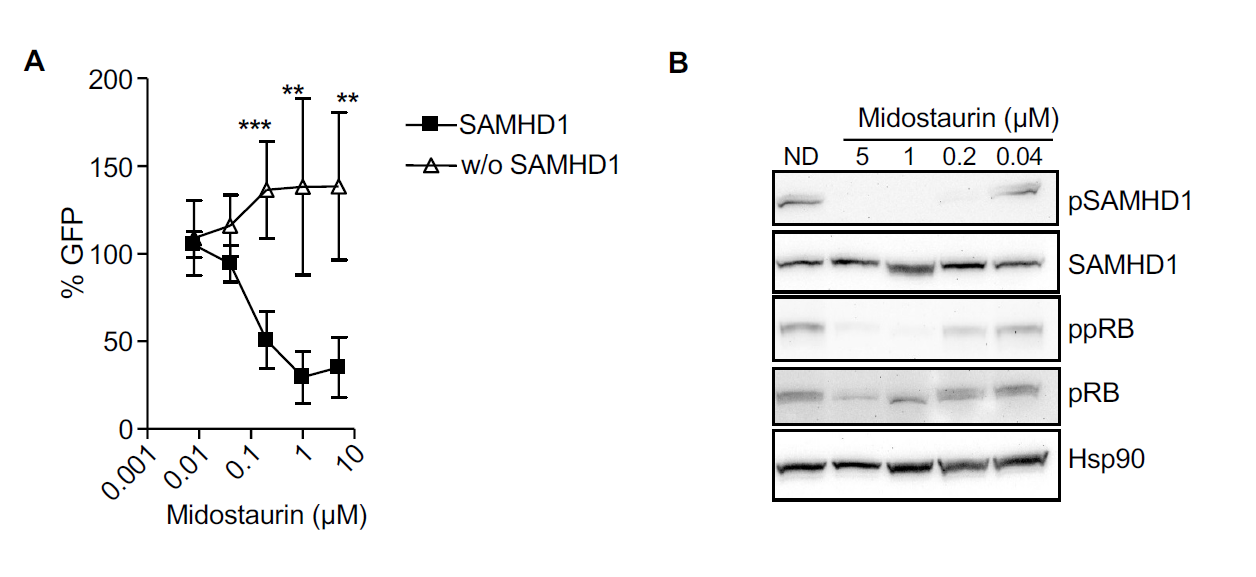


**Figure S1.** (**A**) Efficacy of the multi-kinase inhibitor midostaurin depends on SAMHD1 expression. Dose response of midostaurin, in wild-type (*n*) or SAMHD1-depleted (Δ) MDM. Inhibition of HIV infection was measured as the percentage of GFP+ cells relative to the no drug condition. Mean ±SD of at least three independent donors performed in. duplicate is shown. (**B**) Midostaurin blocks SAMHD1 inactivation by phosphorylation. Western blot analysis of lysates of untreated MDM (no drug, ND) or macrophages treated with midostaurin at the indicated doses. Membranes were blotted with an anti phospho- SAMHD1 antibody, total SAMHD1, anti phosho-pRB and total pRB. Hsp90 antibody was. used as control. A representative donors is shown.


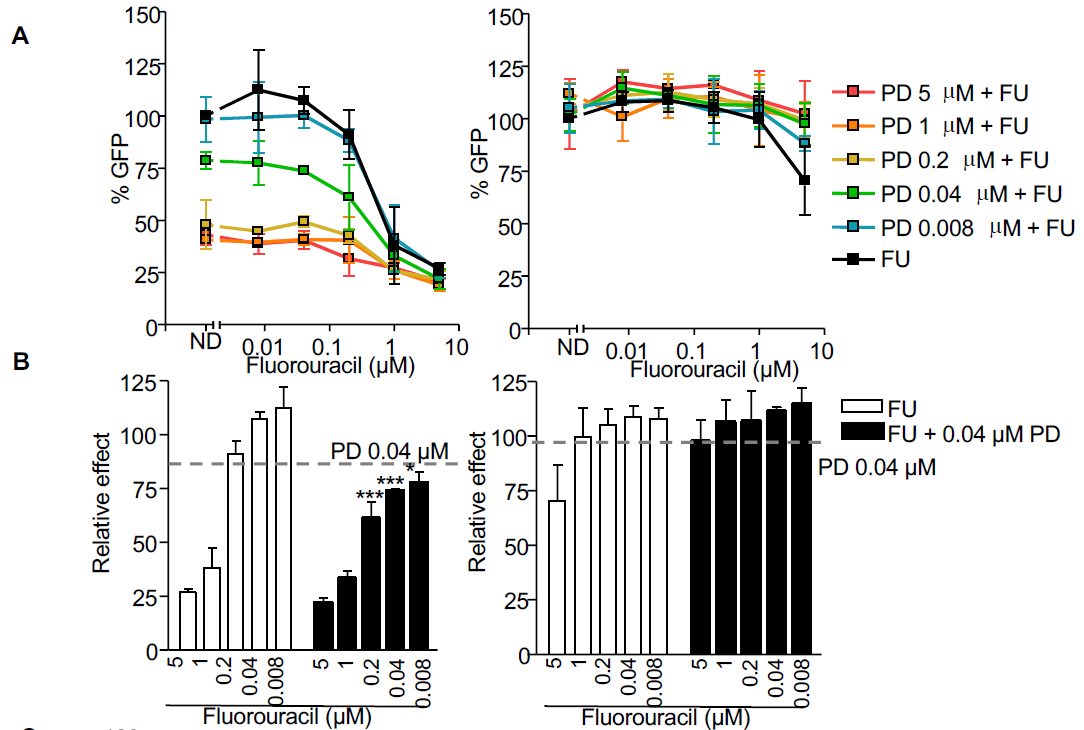


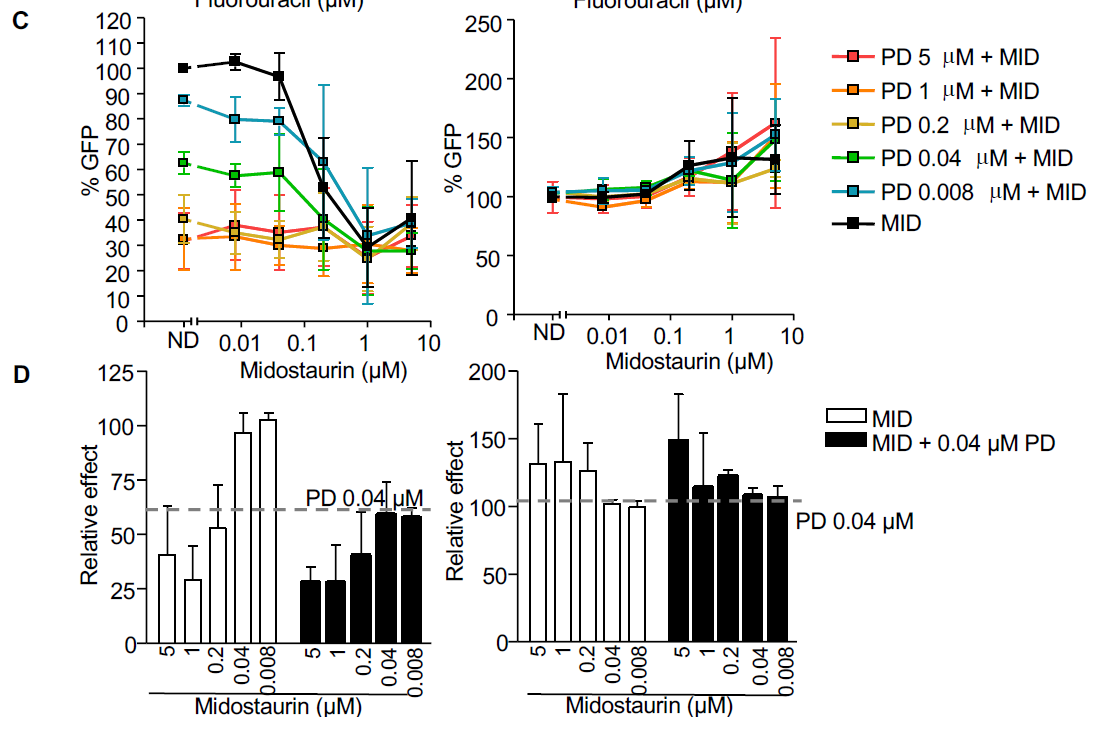


**Figure S2.** (**A**) Relative effect of the combination of palbociclib-fluorouracil measured as antiviral activity. Inhibition of HIV infection with increasing doses of palbocicliband fluorouracil (FU) was measured. Percentage of GFP+ cells relative to the no drug condition. is shown in presence (left panel) or absence (right panel) of SAMHD1. Mean ±SD of at leastthree independent donors performed in duplicate is shown. (**B**) As in (**A**) Relative effect offluorouracil alone (white bars) or in combination with a fixed dose of palbociclib 0.04 μM (black. bars), in the presence (left panel) or absence (right panel) of SAMHD1.Mean ±SD of at leastthree independent donors performed induplicate is shown. (**C**) Relative effect of thecombination of palbociclibmidostaurin measured as antiviral activity. Inhibition of HIV infectionwith increasing doses of palbociclib and midostaurin was measured. Percentage of GFP+ cellsrelative to the no drug condition is shown in presence (left panel) or absence (right panel) ofSAMHD1. Mean ±SD of at least threeindependent donors performed in duplicate is shown. (**D**) As in (**C**) Relative effect of midostaurin alone (white bars) or in combination with a fixeddose of palbociclib 0.04 μM (black bars), in the presence (left panel) or absence (right panel) ofSAMHD1. Mean ±SD of at least three independent donors performed in duplicate is shown.


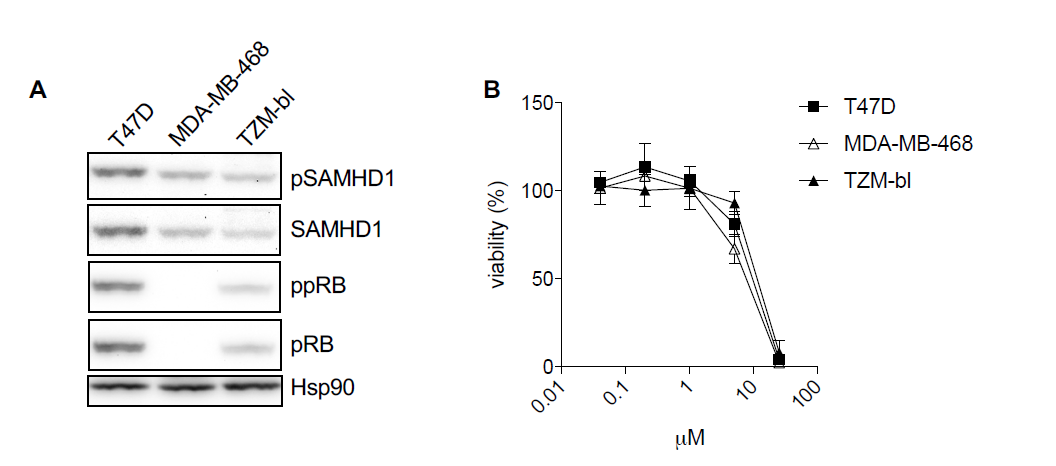


**Figure S3.** (**A**) Protein expression in the different cell lines used. Western blot showing expression and phosphorylation of pRB and SAMHD1 in T47D, MDA-MB-468 and TZMbl cell lines. Membranes were blotted with an anti phospho-SAMHD1 antibody, total SAMHD1, anti phosho-pRB and total pRB. Hsp90 antibody was used as control. A representative experiment is shown. (**B**) Cytotoxic activity of palbociclib in cell lines. Cell viability was measured after palbociclib treatment, observing a clear dose-response. Mean ±SD of at three independent experiments performed in triplicate is shown.

**Table S1.** Cytotoxic activity of drugs evaluated in the different cell lines. CC50 of the different compounds were calculated in TZM-bl, MDA-MB-468 and T47D cells. Cytotoxic effect of the drugs was tested by MTT assay.


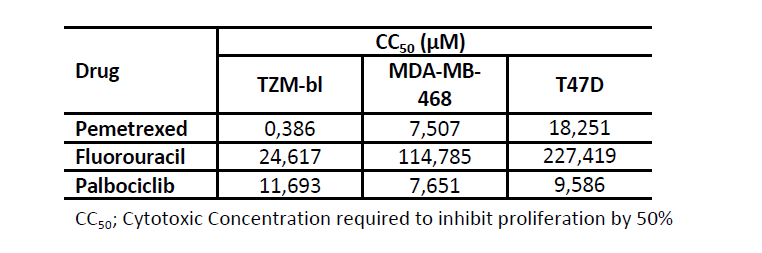


CC50; Cytotoxic Concentration required to inhibit proliferation by 50%.


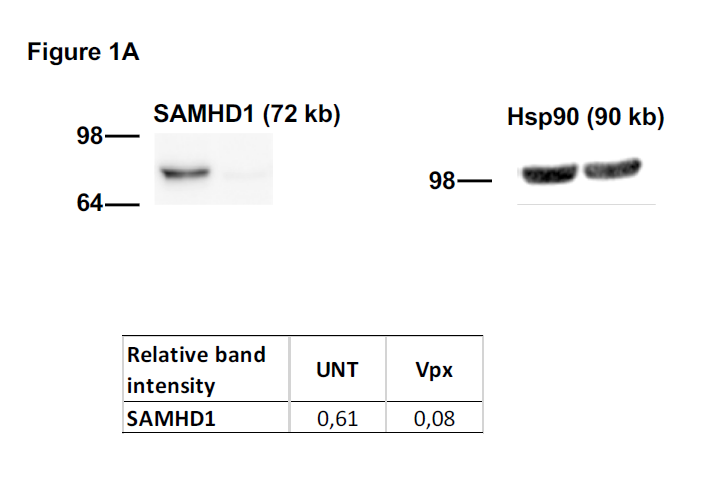


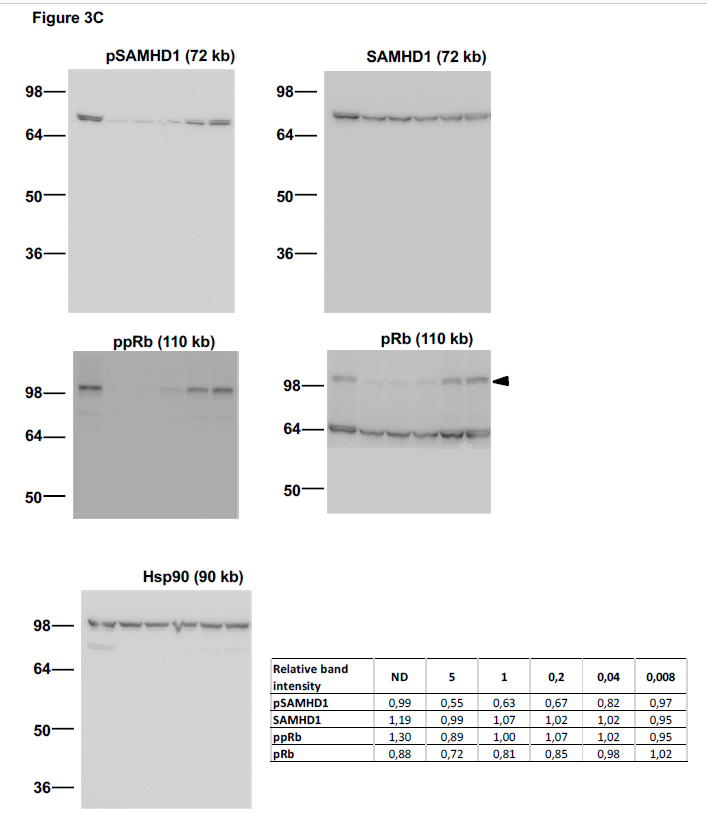


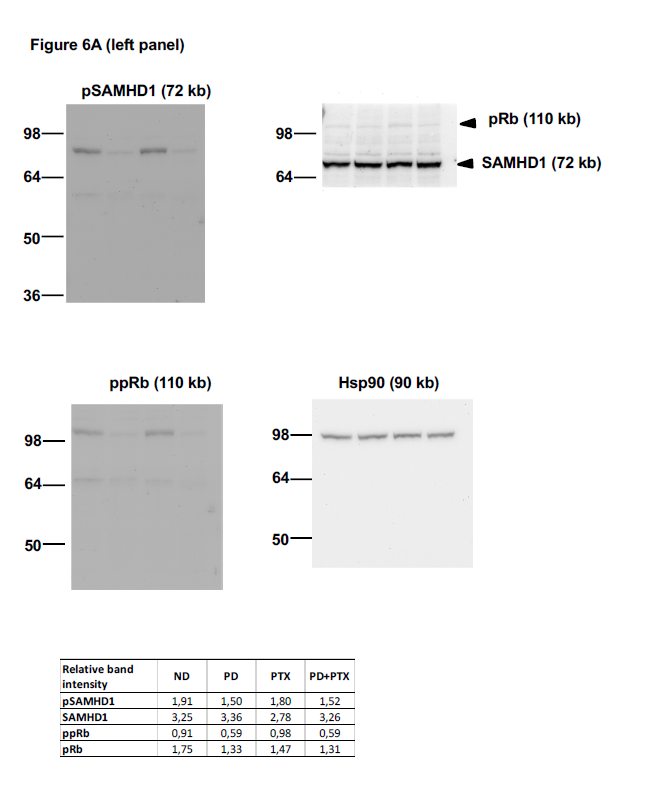


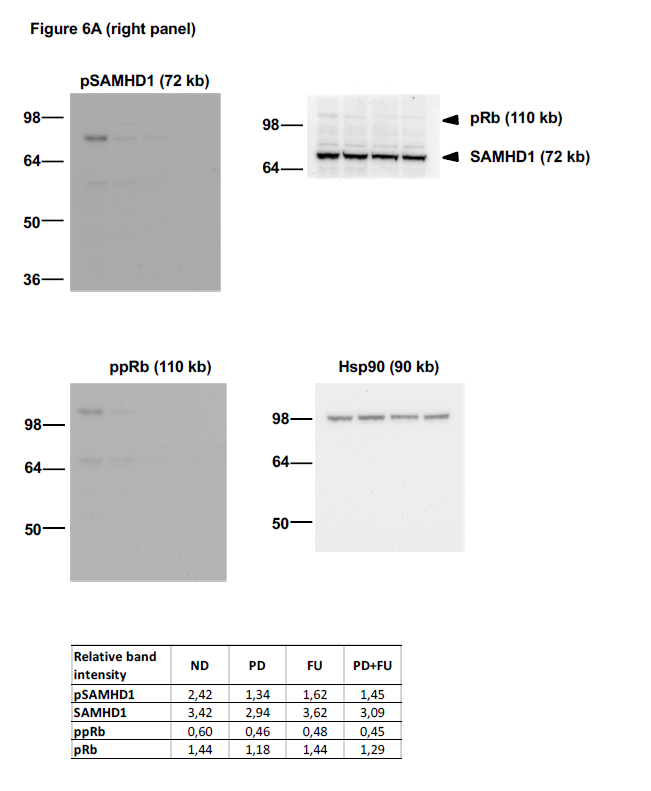


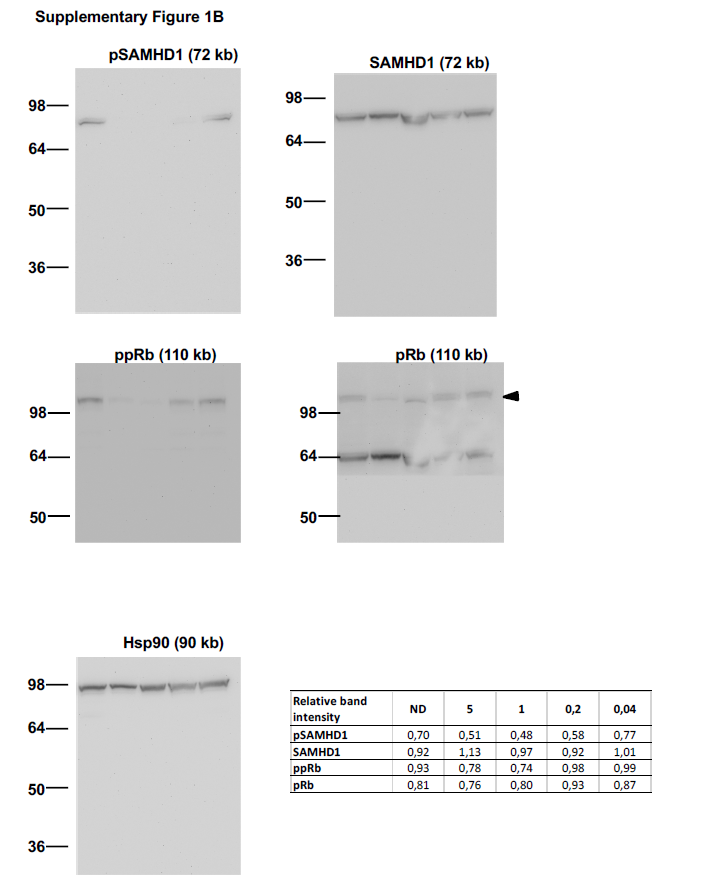


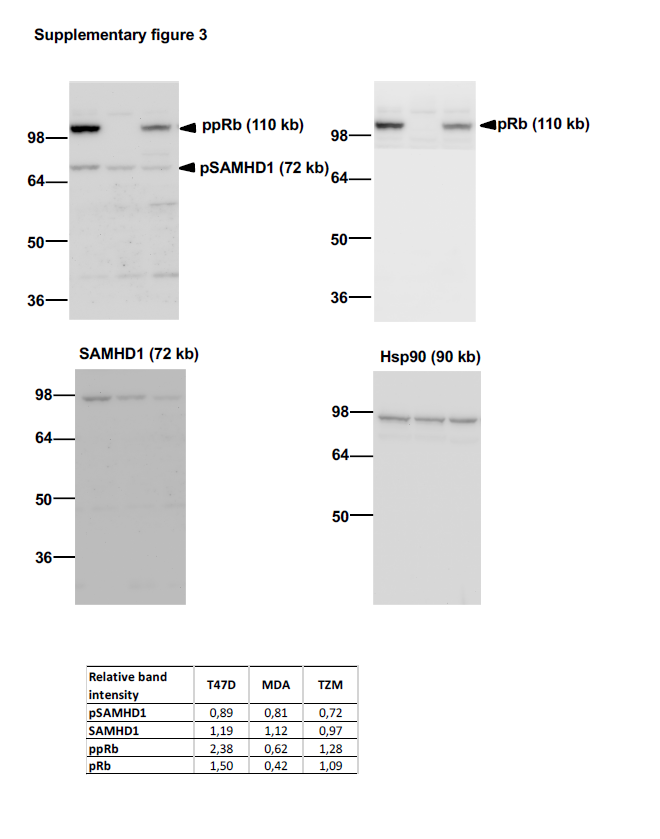


| 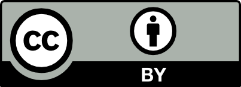 | © 2020 by the authors. Licensee MDPI, Basel, Switzerland. This article is an open access article distributed under the terms and conditions of the Creative Commons Attribution (CC BY) license (http://creativecommons.org/licenses/by/4.0/). |
| --- | --- |
